# Evaluation of Caries Detection on Bitewing Radiographs: A Comparative Analysis of the Improved Deep Learning Model and Dentist Performance

**DOI:** 10.1111/jerd.13470

**Published:** 2025-04-07

**Authors:** Baturalp Ayhan, Enes Ayan, Gökhan Karadağ, Yusuf Bayraktar

**Affiliations:** ^1^ Department of Restorative Dentistry, Faculty of Dentistry Bursa Uludag University Bursa Turkey; ^2^ Department of Computer Engineering, Faculty of Engineering and Architecture Kırıkkale University Kırıkkale Turkey; ^3^ Department of Restorative Dentistry, Faculty of Dentistry Kırıkkale University Kırıkkale Turkey

**Keywords:** artificial intelligence, caries detection, deep learning, dental radiographs, YOLO

## Abstract

**Objectives:**

The application of deep learning techniques for detecting caries in bitewing radiographs has gained significant attention in recent years. However, the comparative performance of various modern deep learning models and strategies to enhance their accuracy remains an area requiring further investigation.

**Methods:**

This study explored the capabilities of 11 widely used YOLO (You Only Look Once) object detection models to automatically identify enamel and dentin caries from bitewing radiographs. To further optimize detection performance, the YOLOv9c model's backbone architecture was refined, reducing both model size and computational requirements. The enhanced model was assessed alongside six dentists, using the same test dataset for direct comparison.

**Results:**

The proposed YOLOv9c model achieved the highest performance among the evaluated models, with recall, precision, specificity, F1‐score, and Youden index values of 0.727, 0.651, 0.726, 0.687, and 0.453, respectively. Notably, the YOLOv9c model surpassed the performance of the dentists, as indicated by its recall and F1‐score values.

**Conclusions:**

The proposed YOLOv9c model proved to be highly effective in detecting enamel and dentin caries, outperforming other models and even clinical evaluations by dentists in this study. Its high accuracy positions it as a valuable tool to augment dentists' diagnostic capabilities.

**Clinical Significance:**

The results emphasize the potential of the YOLOv9c model to assist dentists in clinical settings, offering accurate and efficient support for caries detection and contributing to improved patient outcomes.

## 1 Introduction

Dental caries represents a significant public health concern that causes most dental pain and tooth loss. Considering their preventable and treatable nature, dental caries remains an extensive issue. The thorough and early detection of dental caries has critical importance for the well‐timed and effective administration of treatment [1]. Substantial tooth cavities induced by dental caries can be easily detected by visual‐tactile method. However, these assessments provide limited information about the extent of the caries lesion and may be insufficient for caries detection [2]. In addition to clinical examination, radiographic assessment, especially bitewing radiography, is the most preferred method to detect caries lesions, especially approximal caries, and to evaluate their depth and change [3]. Despite careful examination of dental radiographs, the diagnostic accuracy of approximal caries depends on the expertise of the examining dentists [4]. It has been demonstrated that less experienced dentists exhibit reduced accuracy compared to more experienced counterparts; dentists with more experience have an up to nearly four times greater possibility of correctly detecting approximal caries than those with limited experience [5]. In addition to experience, misdiagnosis or diagnostic inaccuracies may occur in high‐volume clinics due to the distraction of dentists. The implementation of computer‐aided systems to prevent situations can reduce the workload of dentists and increase the possibility of success [6]. As artificial intelligence (AI) continues to develop at a rapid pace, the possibility of creating an automated caries detection system with increased efficiency and precision has become a realistic prospect. This has the potential to significantly enhance the process of clinical caries diagnosis [7]. Initially, researchers used classical unsupervised models or shallow neural networks to detect caries in dental X‐rays, relying on traditional image processing or manual feature selection, both of which limited adaptability and feature integrity [8, 9]. Over the past few years, researchers have constantly revealed the employment of deep learning (DL) with convolutional neural networks (CNNs) to process diverse types of radiographic images, with promising outcomes [10]. In these studies, numerous conditions and clinical situations were evaluated using CNNs in dentistry, including tooth detection and numbering [11, 12], caries detection [11, 13, 14], assessment of tooth morphology [15, 16], periapical lesion detection [17, 18], periodontal bone loss detection [19, 20], root fracture [21, 22], mandibular canal and impacted tooth detection [23, 24], jaw lesion detection [25, 26], implant detection and classification [27, 28], and cephalometric landmark detection and analysis [29, 30].

Advancements in DL have emerged as valuable tools in the healthcare sector, offering significant potential to assist experts and provide second opinions in daily clinical practice [31]. The utilization of AI within the domain of dentistry entails the analysis of complex data, which is then utilized to formulate personalized treatment strategies. This approach facilitates the delivery of care that is more precise and efficacious for each individual patient [32]. In this context, progress in image processing and computer vision has propelled the YOLO (You Only Look Once) series to the forefront of real‐time object detection. YOLO algorithms, esteemed for their exceptional speed and accuracy, have been extensively utilized across a wide range of domains, including healthcare and dentistry. The latest version, YOLOv9 [33], presents notable improvements; however, challenges remain in balancing model size, processing speed, and accuracy, particularly in reducing computational demands while maintaining or enhancing performance. Evaluating the performance of these continuously evolving models on current clinical challenges is crucial to ensure their suitability for real‐world applications.

In line with this objective, the aim of this study is to develop a DL‐based CNN model to detect approximal dental caries by categorizing them into enamel and dentin caries on bitewing radiographs, and to compare the performance with the outcomes of dentists who undergo specialized training. The main contributions of this study are summarized below.

- Eleven different YOLO models were trained to detect approximal caries on bitewing radiographs and classify them as enamel or dentin caries. A comprehensive performance comparison was then conducted.
- By improving the backbone of the YOLOv9 model, an increase in detection performance was achieved while reducing the model size and computational cost.
- The performance of the developed model in detecting approximal caries on bitewing radiographs has been thoroughly evaluated against that of dentists.

## 2 Materials and Methods

### 2.1 Dataset

This study was conducted at the Department of Restorative Dentistry, Faculty of Dentistry, Kırıkkale University, and approved by the non‐interventional Clinical Research Ethical Committee of Kırıkkale University (Date: 31.01.2024, Decision Number: 2024.01.27). Bitewing radiographic image datasets that comprise no personal information, acquired between January‐2018 and January‐2024, were obtained from the radiology archive of the university. Bitewing radiographs were acquired using a radiographic device (Gendex Dental Systems, IL, USA) with a 0.04 mm focal spot, 65 kVp tube voltage, and 7 mA tube current. Due to variations in acquisition protocols across departments and personnel, variations in exposure time, distance, and resultant image quality were likely.

**FIGURE 1:**
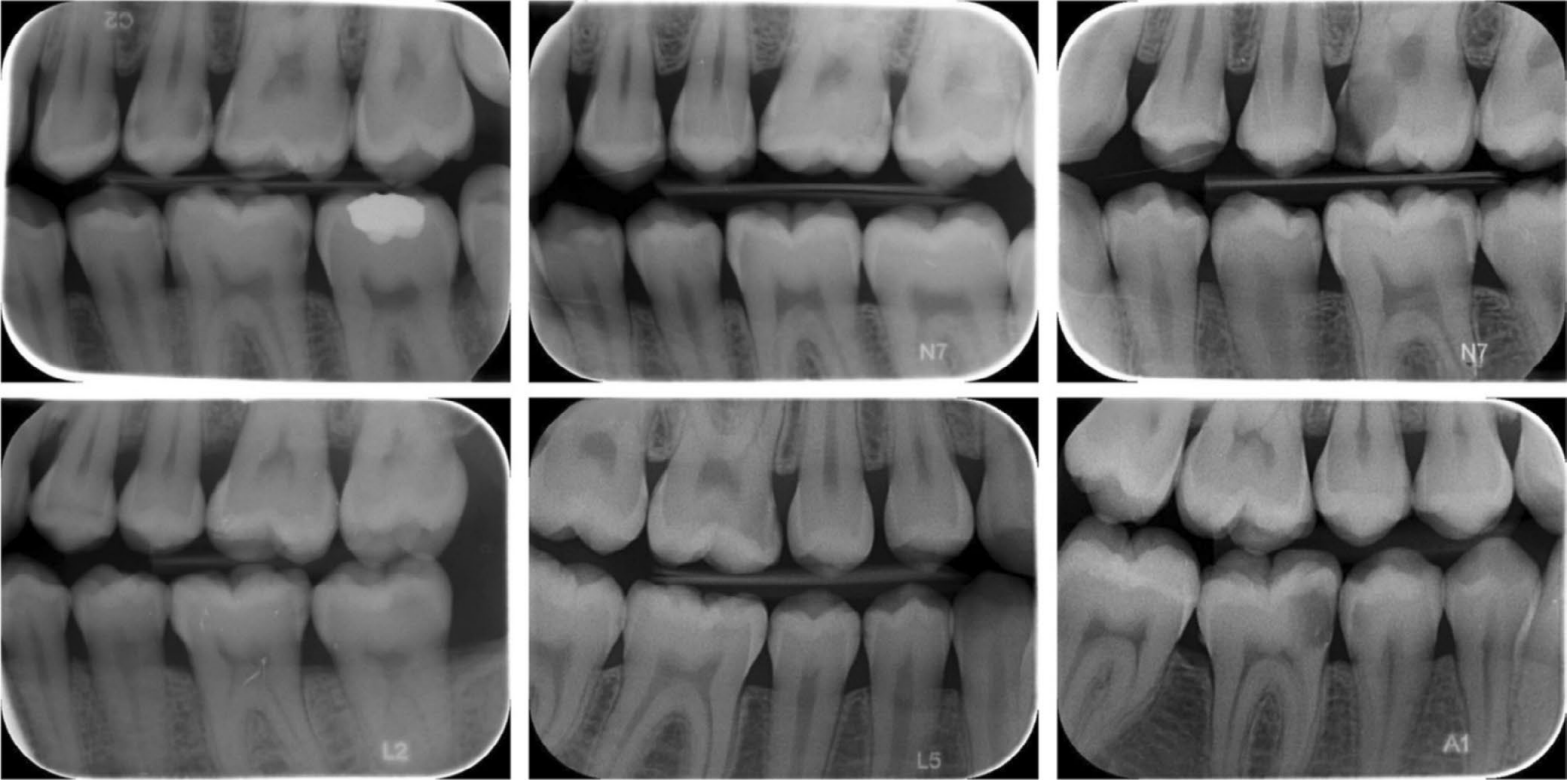
Bitewing samples from dataset.

Each radiographic image that has been anonymized has been saved in JPEG format. The evaluation was conducted on the proximal surfaces of the posterior teeth in the maxillary and mandibular dental arches, as visualized in the radiographic images. Images that contain technical or patient positioning errors that could complicate or completely prevent diagnosis were not included in the dataset. Third molars were excluded from the analysis due to their inability to fit entirely within the radiographic image and their low prevalence. In addition, approximal surfaces with restorations were not included in the analysis because it could not be determined from the radiographic image whether any underlying caries were residual or secondary. A total of 2150 bitewing radiographs were collected for this study and subsequently divided into two subsets: a training and validation set of 2000 images and a testing set of 150 images. The training dataset includes 14,800 teeth, 29,380 approximal surfaces, and 8096 caries labels, categorized as 2491 enamel caries and 5605 dentin caries. The details of the dataset are presented in Table 1, while sample images from the dataset are shown in Figure 1.

**TABLE 1:** Dataset details.

	Train	Test
Decay‐overall	8096	891
Decay‐enamel	2491	386
Decay‐dentin	5605	505
Teeth‐overall	14,800	1170
Surfaces‐overall	29,380	2160
Total image	2000	150

### 2.2 Study Design

**FIGURE 2:**
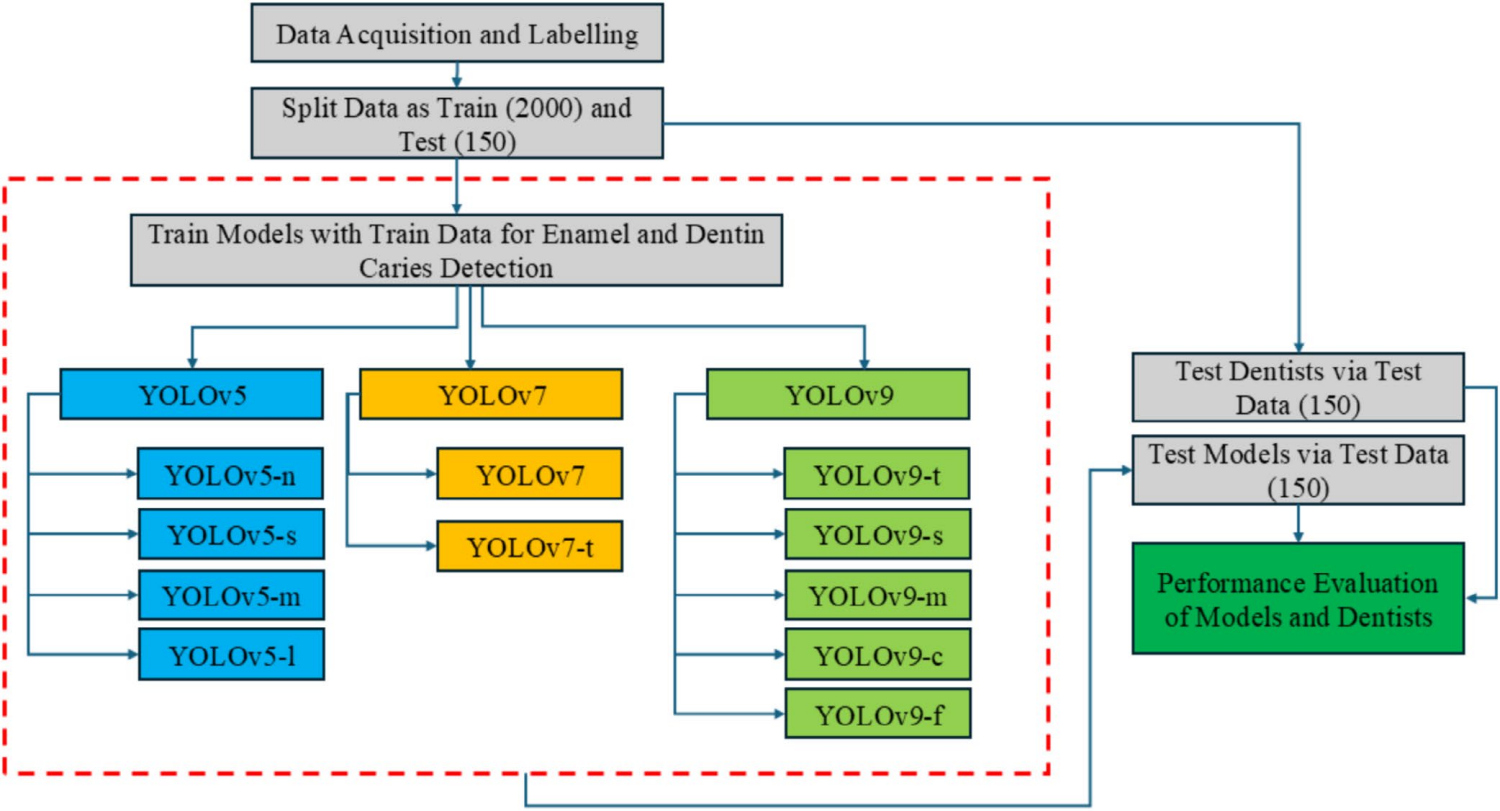
Study design.

This study aims to modify the backbone of the YOLOv9 model to develop a lightweight yet more effective architecture, referred to as YOLOv9c‐Faster. The revised model aims to establish a better balance between computational efficiency and accuracy in the detection of dental caries from bitewing radiographs. The performance of YOLOv9c‐Faster is comprehensively evaluated against the original YOLOv9, as well as previous versions YOLOv7 and YOLOv5. To further assess the effectiveness of the developed model, caries detection performance has been compared with the evaluations from six different dentists. Ultimately, the goal of this research is to propose a more efficient model suitable for real‐world applications, particularly in the field of medical imaging. The study design is shown in Figure 2.

### 2.3 Image Evaluation and Data Labeling

A restorative specialist (author G.K.) with 12 years of clinical experience and a research assistant (author B.A.) with 6 years of clinical experience conducted a comprehensive discussion and then meticulously labeled the caries lesions as enamel and dentin using a software program developed in the Python programming language. Thereafter, a restorative specialist (author Y.B.) with 15 years of clinical experience assessed and revised these labels, which were then recreated as the definitive ground truth labels. For labeling bounding boxes, annotators provided a class label for each box indicating the presence of enamel and dentin caries. A collective of six dentists, all of them employed at the same university's clinical department for a minimum of 1 year and a maximum of 5 years, was used as a comparator group to enable the estimation of the relative performance of the DL models in comparison to individual dentists. Each of the dentists completed the labeling task on a set of 150 radiographic images, from which 144 images showed 386 enamel caries and 505 dentin caries; six images were negative cases. It is noteworthy that no specific training on caries detection was provided to the dentists participating in this study. Prior to undertaking the labeling task, all participating dentists underwent training in the use of the labeling program.

### 2.4 You Only Look Once (YOLO)

Object detection is a critical problem that needs to be addressed in computer vision. In order to solve this problem, researchers from the past to the present have developed a variety of algorithms [34]. In recent years, with the popularization of DL algorithms, CNN‐based object detection algorithms have emerged. One of these algorithms, YOLO, stands out from other algorithms due to its real‐time object detection speed and small model size. The first version of YOLO was introduced by Redmon et al. [35]. Unlike two‐stage object detection algorithms [36], YOLO performs both bounding box localization and classification in a single step.

YOLO's framework segments the image into uniform grids and facilitates end‐to‐end learning by predicting bounding boxes and class probabilities simultaneously for each grid [35]. YOLO is a highly adaptable model, and its real‐time detection efficiency has transformed fields such as medical imaging [37], autonomous vehicles [38], security systems [39], and industrial production [40]. In these fields, both accuracy and speed are of paramount importance. Over time, multiple versions of YOLO have been presented, ranging from YOLOv2 to YOLOv11 [33, 41, 42, 43, 44, 45, 46]. In this study, we evaluated different versions of YOLOv5, YOLOv7, and YOLOv9 architectures, along with our improved version of YOLOv9c‐Faster.

#### 2.4.1 YOLOv5

YOLOv5 was introduced in 2020 by Glenn Jocher et al. [43]. Similar to other YOLO models, its architecture consists of input, backbone, neck, and head (output) modules. The model accepts images with dimensions of 640 × 640 or 1280 × 1280 as input. Unlike its predecessors, which utilized the Darknet framework, YOLOv5 was developed using the PyTorch framework [47], increasing accessibility for researchers. Based on the number of parameters, different versions of architecture have been proposed, ranging from smaller to larger models: v5n, v5s, v5m, v5l, and v5x. The current study evaluates the performance of the YOLOv5n, YOLOv5s, YOLOv5m, and YOLOv5l models in detecting caries in bitewing radiographs.

#### 2.4.2 YOLOv7

YOLOv7 was introduced in 2022 by Chien‐Yao Wang and his team [44]. The model architecture incorporates significant improvements in the backbone network, activation function, and loss function. The improvements enable the model to achieve better classification speed and performance while using 40% fewer parameters compared to other object detection algorithms. Similar to earlier YOLO versions, YOLOv7 is composed of input, backbone, neck, and head modules, and it supports input image sizes of 640 × 640 or 1280 × 1280. A key innovation in YOLOv7 is the incorporation of the Extended Efficient Layer Aggregation Network (E‐ELAN) in its neck design. The E‐ELAN module enhances the network's learning capabilities by employing expand, shuffle, and merge connections to optimize learning without disrupting the original gradient flow. YOLOv7 comes in various versions, including YOLOv7‐tiny, YOLOv7, and YOLOv7‐w6. In the current study, the YOLOv7‐tiny and YOLOv7 models were evaluated in detecting caries in bitewing radiographs.

#### 2.4.3 YOLOv9

The YOLOv9 model was introduced in 2024 by Chang et al. [33]. It represents one of the most recent iterations of the YOLO architectural framework. The model introduces two innovative features to achieve greater accuracy: the first is the Generalized Efficient Layer Aggregation Network (GELAN) and the second is Programmable Gradient Information (PGI). The purpose of PGI is twofold: firstly, to prevent data loss, and secondly, to ensure precise gradient updates. In contrast, GELAN is employed to optimize lightweight models through gradient path planning. It has been shown that combining PGI with the adaptive GELAN architecture improves the model's learning capabilities. At the same time, it ensures the preservation of crucial information throughout the detection process. YOLOv9 is available in four variants, categorized based on the number of parameters: v9s, v9m, v9c, v9l, and v9e. The current study evaluates the performance of the YOLOv9s, YOLOv9m, and YOLOv9c models in detecting caries in bitewing radiographs.

### 2.5 FasterNet Architecture

**FIGURE 3:**
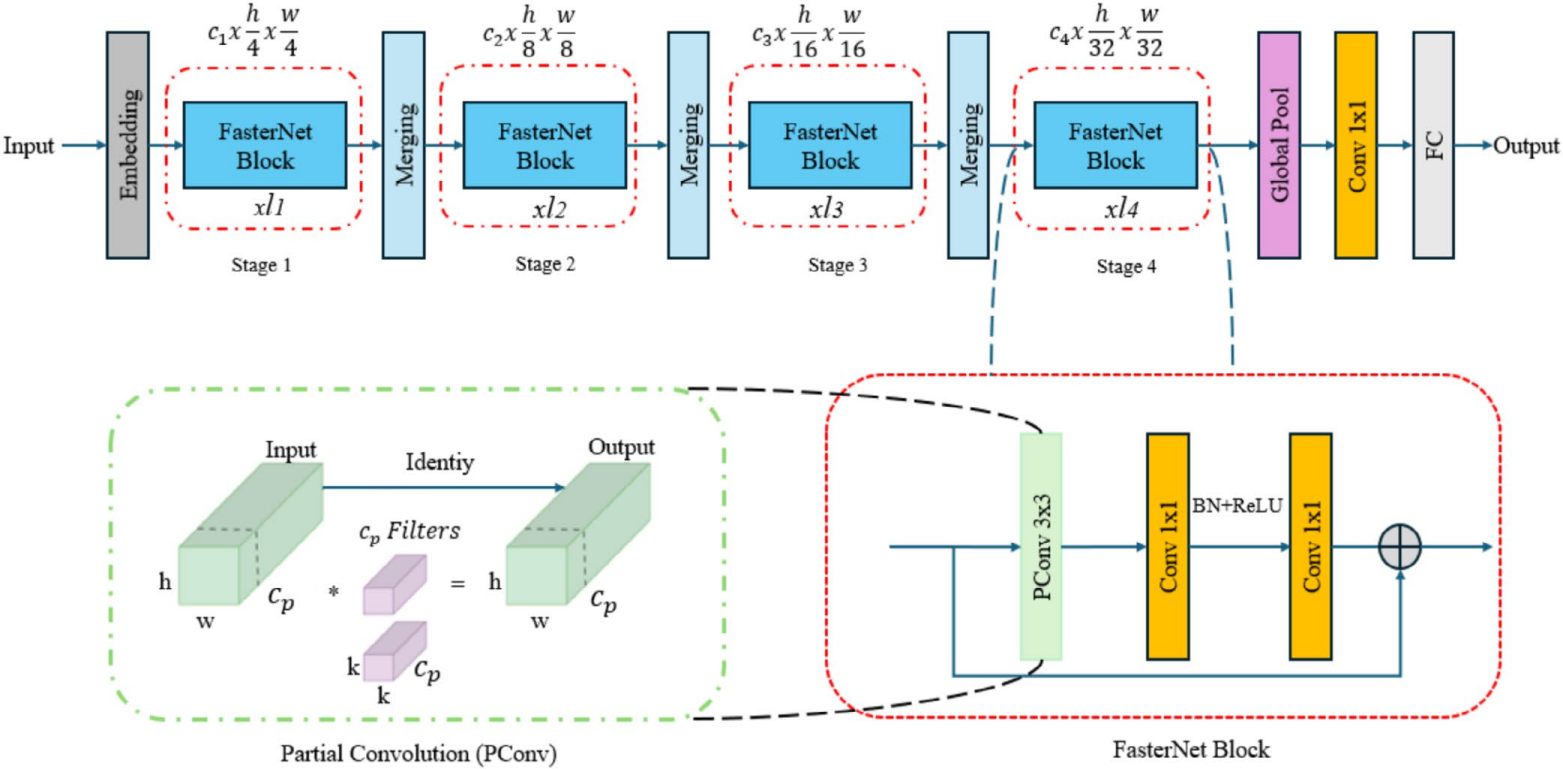
FasterNet architecture details.

FasterNet is an efficient backbone network designed for object detection tasks by Chen et al. [48]. The model employs an innovative operator, designated as PConv (Partial Convolution), which is designed to optimize the balance between speed and accuracy. This operator processes only a subset of input channels, thereby avoiding unnecessary computations and memory access. The utilization of PConv serves to enhance computational efficiency while simultaneously increasing the number of operations per second, thereby resulting in a notable improvement in performance. FasterNet is comprised of four hierarchical stages, each containing PConv layers and other convolutional layers to ensure efficient computation. FasterNet architecture details are given in Figure 3.

This structure enables the detection of micro‐targets with high precision in complex environments. Additionally, FasterNet performs well across different processors, making it suitable for use in mobile and embedded devices. The model has been evaluated across multiple computer vision tasks, such as object detection, classification, and image segmentation. The PConv module is the main novelty of FasterNet. It performs standard convolution on only subsets of the input channels, leaving the rest unchanged to minimize unnecessary computations and memory access. The specifics of PConv's computational and memory access processes are as follows:

(1) $${\text{FLOPs}}_{\text{PConv}}=h\times w\times k^{2}\times c_{p}^{2}$$

(2) $${\text{MemoryAccess}}_{\text{PConv}}=h\times w\times c_{\mathrm{cp}}+k^{2}\times c_{p}^{2}\approx h\times w\times 2c_{p}$$

In the formulas, *h* and *w* represent the height and width of the feature map, *k* denotes the convolution kernel size, and *c*_*p*_ refers to the total number of sub‐channels used for computation. FasterNet is available in different size variants (Tiny, Small, Medium, Large) designed to address various computer vision tasks, including object detection, classification, and image segmentation. In this study, all variants of FasterNet were assessed, and the most successful architecture, tiny, was employed as the backbone for the YOLOv9c architecture.

### 2.6 Improved YOLOv9 Architecture

**FIGURE 4:**
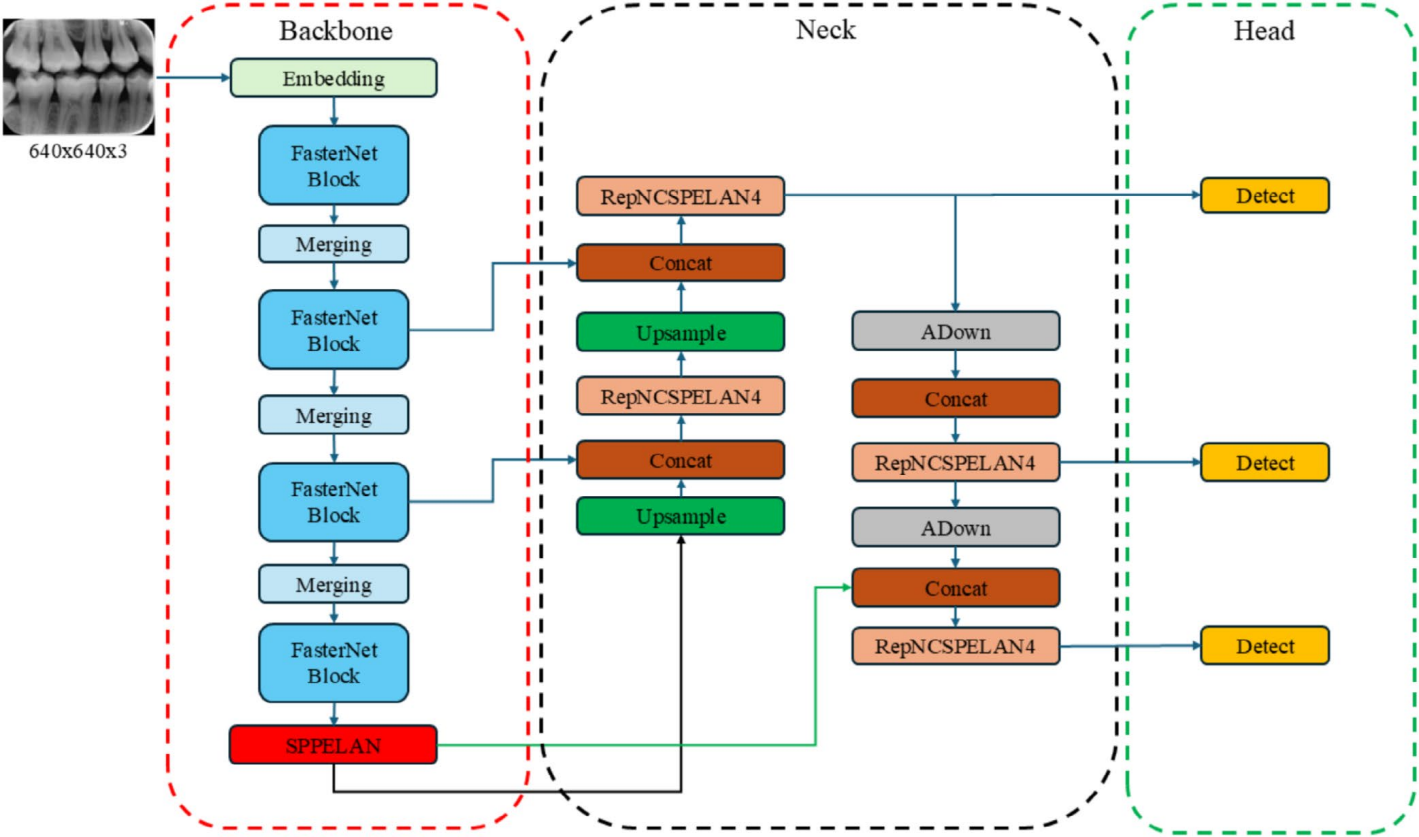
YOLOv9c‐Faster architecture.

In this study, a backbone improvement has been made to the YOLOv9c architecture, which outperforms other models in terms of accuracy. The enhanced YOLOv9c‐Faster network architecture is shown in Figure 4. The improvements in the YOLOv9‐Faster model focus on increasing detection accuracy and speed. This includes the addition of auxiliary training modules designed to better capture the features of interface defects. The model balances computational efficiency with enhanced expressive capability by using the FasterNet network as the backbone [48]. This study presents an adaptive enhancement of the YOLOv9c model, tailored for better performance in interface defect segmentation tasks. A visual representation of the YOLOv9c‐Faster model is provided in Figure 4.

### 2.7 Evaluation Metrics

The caries detection performance of the models and dentists was evaluated using a comprehensive set of metrics, including Precision (P), Recall (R), Specificity (Sp), F1‐score, and Youden index. Additionally, all evaluation metrics were calculated using the micro‐averaging method instead of the macro averaging method [49]. The Youden index, defined as a function of sensitivity and specificity, is widely used to assess overall diagnostic effectiveness. Its values range from 0 to 1, where values near 1 indicate high diagnostic effectiveness of the biomarker, while values approaching 0 reflect limited effectiveness [50]. We adopted the approximal surface level statistical method from the previous research [11]. To be specific, the caries detection outcomes for the approximal surface of a tooth were categorized as true positive (TP), false positive (FP), false negative (FN), and true negative (TN). In the equations, TP represents correctly identified caries, FP represents incorrectly identified caries, FN represents undetected caries, and TN represents non‐carious tooth interfaces. These metrics are calculated according to the following formulas:

(3) $$P=\frac{\mathrm{TP}}{\mathrm{TP}+\mathrm{FP}}$$

(4) $$R=\frac{\mathrm{TP}}{\mathrm{TP}+\mathrm{FN}}$$

(5) $$\mathrm{Sp}=\frac{\mathrm{TN}}{\mathrm{TN}+\mathrm{FP}}$$

(6) $$F1=2\times \frac{P\times R}{P+R}$$

(7) Youden index=R+Sp−1

### 2.8 Development Environment and Training Parameters

The experiments in this study were carried out in a setup featuring Ubuntu 20.04, PyTorch 2.0.0, CUDA 11.8, Python 3.8, an NVIDIA RTX 1080 Ti GPU with 12GB of VRAM, and dual Intel(R) Xeon(R) processors. In order to maintain a consistent comparison, all models were trained using the default training parameters. Transfer learning was utilized during the training process. The training parameters for the models are provided in Table 2.

**TABLE 2:** Training parameters of models.

Parameter name	Value
Input size	640 × 640 × 3
Epochs	200
Batch size	16
Optimizer	Adam
Weight decay	0.0005
Learning rate	0.01
Momentum	0.93
IOU	0.50
Confidence	0.20

## 3 Results

The test dataset, comprising 150 bitewing radiographs, contained 1170 teeth, 2160 approximal surfaces, and 891 caries labels, categorized as 386 enamel caries and 505 dentin caries. Based on the evaluation metrics (TP, FP, FN, TN), caries detection outcomes at the approximal surface level were quantified, after which Recall, Precision, Specificity, F1‐score, and Youden index metrics were calculated. The comparative results for the dentists and models are presented in Tables 3, 4, 5 with the highest and lowest values of each metric highlighted in bold and underlined, respectively.

**TABLE 3:** Micro averaged test results for comparison of YOLOv9c‐Faster and dentists.

		Recall	Precision	Specificity	F1‐score	Youden Index
Dentist	1	0.613	0.680	0.797	0.644	0.410
Dentist	2	0.269	0.740	0.933	0.394	0.202
Dentist	3	0.427	0.670	0.852	0.521	0.279
Dentist	4	0.446	0.772	0.907	0.565	0.353
Dentist	5	0.600	0.799	0.894	0.685	0.494
Dentist	6	0.301	0.719	0.917	0.424	0.218
Model	YOLOv9c‐Faster	0.727	0.651	0.726	0.687	0.453

**TABLE 4:** Detailed test results comparisons for model and dentists.

	D1	D2	D3	D4	D5	D6	YOLOv9c‐Faster
Enamel							
TP	163	81	191	123	201	80	255
FP	95	80	172	84	88	96	245
FN	223	305	195	263	185	306	131
Precision	0.631	0.503	0.526	0.594	0.695	0.454	0.510
Recall	0.422	0.209	0.494	0.318	0.520	0.207	0.660
F1	0.505	0.295	0.509	0.414	0.594	0.284	0.575
Dentin							
TP	384	159	190	275	334	189	393
FP	162	4	15	33	46	9	102
FN	121	346	315	230	171	316	112
Precision	0.703	0.975	0.926	0.892	0.878	0.954	0.793
Recall	0.758	0.314	0.376	0.544	0.661	0.374	0.778
F1	0.729	0.475	0.534	0.675	0.754	0.537	0.786
Overall							
TP	547	240	381	398	535	269	648
FP	257	84	187	117	134	105	347
FN	344	651	510	493	356	622	243
TN	1012	1185	1082	1152	1135	1164	922
Precision	0.680	0.740	0.670	0.772	0.799	0.719	0.651
Recall	0.613	0.269	0.427	0.446	0.600	0.301	0.727
Specificity	0.797	0.933	0.852	0.907	0.894	0.917	0.726
F1	0.644	0.394	0.521	0.565	0.685	0.424	0.687
Youden index	0.410	0.202	0.279	0.353	0.494	0.218	0.453

**TABLE 5:** Detailed test results comparisons for YOLO models.

	5n	5s	5m	5L	7	7t	9t	9s	9m	9c	9c‐Faster
Enamel											
TP	198	165	191	97	215	203	232	218	216	218	255
FP	193	200	197	161	260	198	256	249	237	232	245
FN	188	221	195	289	171	183	154	168	170	168	131
Precision	0.506	0.452	0.492	0.375	0.452	0.506	0.475	0.466	0.476	0.484	0.510
Recall	0.512	0.427	0.494	0.251	0.556	0.525	0.601	0.564	0.559	0.564	0.660
F1	0.509	0.439	0.493	0.301	0.499	0.515	0.530	0.511	0.514	0.521	0.575
Dentin											
TP	381	345	360	331	417	395	394	392	384	388	393
FP	138	121	121	147	142	112	118	106	111	113	102
FN	124	160	145	174	88	110	111	113	121	117	112
Precision	0.734	0.740	0.748	0.692	0.745	0.779	0.769	0.787	0.775	0.774	0.793
Recall	0.754	0.683	0.712	0.655	0.825	0.782	0.780	0.776	0.760	0.768	0.778
F1	0.744	0.710	0.730	0.673	0.783	0.780	0.774	0.781	0.768	0.771	0.786
Overall											
TP	579	510	551	428	632	598	626	610	600	606	648
FP	331	321	318	308	402	310	374	355	348	345	347
FN	312	381	340	463	259	293	265	281	291	285	243
TN	938	948	951	961	867	959	895	914	921	924	922
Precision	0.636	0.613	0.634	0.581	0.611	0.658	0.626	0.632	0.632	0.637	0.651
Recall	0.649	0.572	0.618	0.480	0.709	0.671	0.702	0.684	0.673	0.680	0.727
Specificity	0.739	0.747	0.749	0.757	0.683	0.755	0.705	0.720	0.725	0.728	0.726
F1	0.642	0.592	0.626	0.526	0.656	0.664	0.662	0.657	0.652	0.657	0.687
Youden index	0.388	0.319	0.367	0.237	0.392	0.426	0.407	0.404	0.399	0.408	0.453

The data in Table 3 indicate that YOLOv9c‐Faster reached the highest recall and F1‐score, which are 0.727 and 0.687 respectively, while Dentist‐5 achieved the highest values of 0.799 in precision and 0.494 in the Youden index. Even though YOLOv9c‐Faster had high scores in recall and F1‐score, its precision and specificity were the lowest among the dentists in comparison. Apart from precision and specificity, Dentist‐2 recorded the lowest scores across other evaluation metrics. Regarding the Youden index, YOLOv9c‐Faster, which achieved the highest F1‐score, had the second highest value of 0.453, followed by Dentist‐1.

As shown in Table 4, YOLOv9c‐faster had the highest rates for TP, and it also had the lowest rates for FN across enamel, dentin, and overall. According to Table 5, YOLOv9‐c‐Faster exhibited the highest F1‐score and Youden index values among DL models. The performance ranking from highest to lowest according to the Youden index was as follows: YOLOv9‐c‐Faster > 7t > 9c > 9t > 9s > 9m > 7 > 5n > 5m > 5s > 5L. According to the F1‐score, the order was: 9c‐faster > 7t > 9t > 9s = 9c > 7 > 9m > 5n > 5m > 5s > 5L. The improved YOLOv9c‐Faster model accomplished well in caries detection on bitewing radiographs.

## 4 Discussion

Bitewing radiography is a very useful diagnostic method in the diagnosis of approximal caries lesions [51]. As well as the rapid advancement of DL has significantly stimulated interest within the field of dentistry, leading to a substantial increase in investigations into its potential applications for caries detection. However, many dentists lack the requisite knowledge about DL‐based systems and are uncertain about their application and effectiveness in caries detection, particularly when compared to the diagnostic competence of experienced dentists. This study aims to systematically evaluate and compare the caries detection performance of various DL algorithms, including a custom‐developed model by our research team, against that of clinical dentists. Key metrics such as Recall and Specificity were assessed for both the models and the dentists using a bitewing dental X‐ray dataset. Additionally, the Youden index was employed to provide a comprehensive measure of diagnostic performance. The developed YOLOv9c‐Faster model exhibited superior performance over other models, achieving an F1 score of 0.687, a Recall of 0.727, and a Youden index of 0.453 across all classes. The YOLOv9c‐Faster model also demonstrated remarkable success in distinguishing enamel and dentin lesions. The model identified 255 instances of enamel lesions as TPs, achieving an F1 score of 0.575, a Precision of 0.510, and a Recall of 0.660. With regard to enamel caries, the model demonstrates greater success than both dentists and other models in terms of the number of TPs identified. In the case of dentin lesions, the model identified 393 TPs and achieved a Precision of 0.793, a Recall of 0.778, and an F1 score of 0.786. In the case of dentin caries, the model demonstrated greater success than both dentists and other models in terms of the number of TPs identified. The results obtained demonstrate that the model exhibits a high level of efficacy in detecting lesions of both types. These values demonstrate that the model demonstrates consistent and superior performance compared to that of dentists in the detection of enamel and dentin lesions. Nevertheless, its performance is observed to be inferior, particularly in enamel detection, in comparison to dentin. This is due to the fact that enamel caries lesions are less prevalent in the total number of samples used for training in the dataset, and that accurately detecting this type of caries lesion is a challenging process. To address this issue, measures such as data augmentation and dataset expansion should be implemented. While keeping the FP rate under control is crucial for a model to be reliable and effective in clinical settings, the FN rate is of greater importance in medical classification problems. It is less critical for a model to mistakenly label a healthy surface as carious or misidentify caries, compared to missing an actual carious lesion. Ultimately, the results can be easily verified through clinical examination.

**FIGURE 5:**
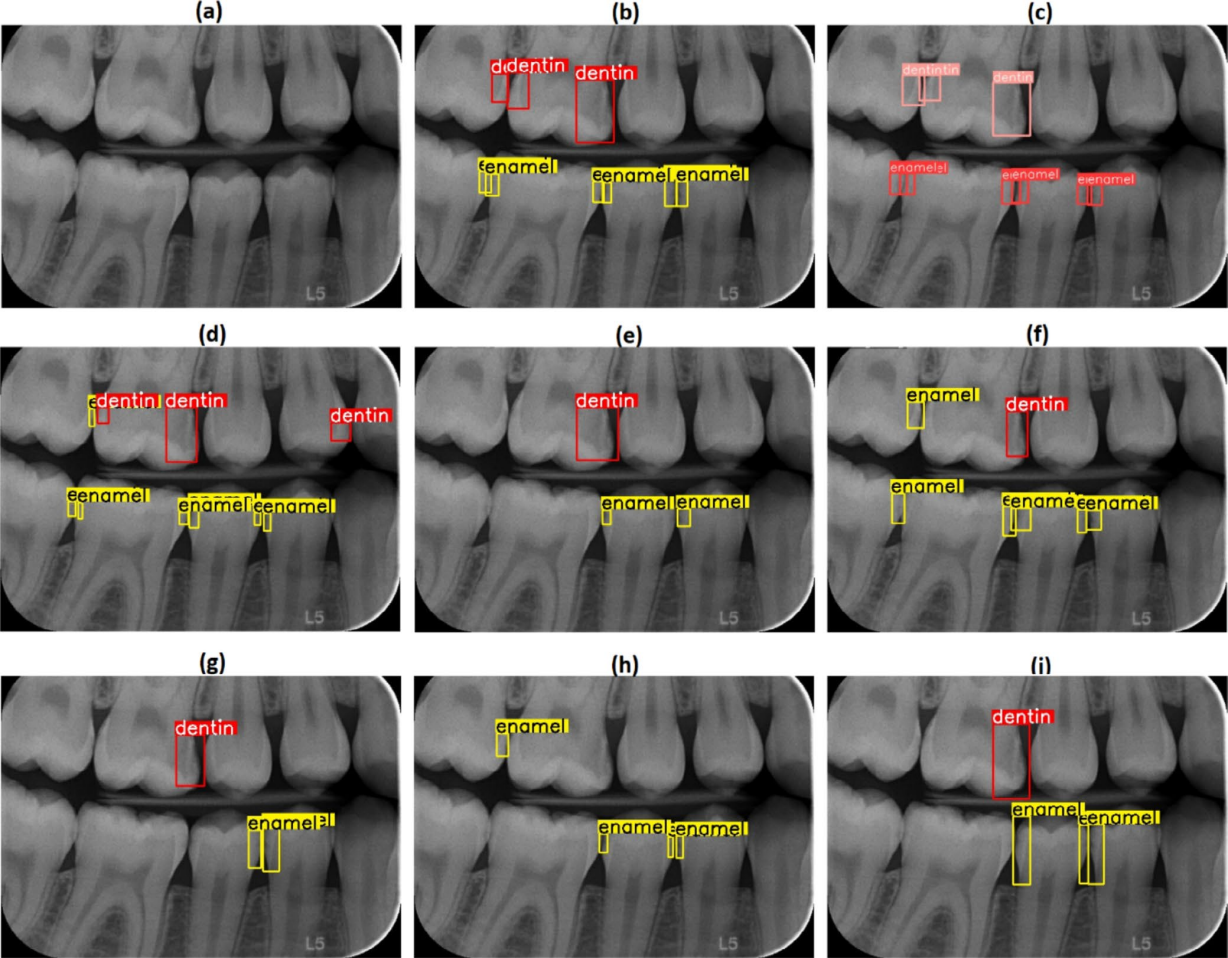
(a) original image, (b) ground truth, (c) model predictions, (d), (e), (f), (g), (h), (i) represent d1, d2, d3, d4, d5, and d6 predictions respectively.

Prior research has shown a positive correlation between dentists' experience and their diagnostic accuracy in caries detection [4, 5]. The findings of the current study, which examined dentists with 1–5 years of experience, align with previous research. However, it was observed that the performances of dentists differed depending on experience and fatigue. For example, Dentist‐5 showed the highest Precision value with 0.799, while its Recall value fell behind the YOLOv9c‐Faster model with 0.600. Dentist‐2 was determined to have the lowest performance with an F1 score of 0.394 and a Recall value of 0.269. This situation suggests that fatigue may be effective, especially in environments where large data sets are used. On the other hand, DL‐based models were not affected by such external factors. It processed 150 images in 15 s. The most challenging images in the test data set and the model with the best detection performance, the dentist, and the required values are given in Figure 5.

In recent years, CNNs have been utilized for diagnostic purposes in dentistry, leveraging various types of dental images and demonstrating promising results [14, 52, 53, 54]. It was reported in a previous study that the CNN‐based YOLOv3 model has shown successful results with high accuracy scores in terms of diagnosing approximal caries lesions from bitewing radiographs [55]. Whereas a previous study has focused on the evaluation of a single model, the current investigation compared the performance of 11 different models, subsequently comparing the results of the optimal model with those obtained by dentists. Thus, a more comprehensive comparative study of DL‐based models was conducted.

Earlier studies have shown that the detection rates for initial‐stage enamel caries, which are notably challenging to identify even in clinical practice, were reported to be slightly lower than those for dentin caries [56, 57]. In the current study, it was also observed that novice dentists demonstrated lower detection performance compared to their more experienced counterparts, and the model achieved results closely aligned with those of the experienced ones.

The quality and scope of the dataset are critical factors affecting model performance. The 2150 bitewing radiographs used in this study were selected in accordance with quality standards. However, it should be noted that in real clinical practice, radiographs may vary in terms of quality, exposure, and artifacts. This diversity can test the generalization capacity of DL‐based models. In particular, the inclusion of images obtained from different radiography devices and restored teeth in the dataset will enable the model to find a wider area of use in real‐world scenarios. There are limitations to the study. In conclusion, this study demonstrates the potential of the DL‐based YOLOv9c‐Faster model to improve dental diagnostic processes. The model showed superior performance in the detection of both enamel and dentin lesions. However, in order to use these technologies effectively, it is necessary to focus on data diversity, general validity, and clinical integration processes. However, deep neural networks should only be used as an auxiliary tool, and the final decision should always be made by the dentist.

In addition, the study compared the detection performance and size of the models. As evidenced in Table 6, our proposed model outperformed YOLOv9‐c and YOLOv5‐l models, despite having fewer parameters. Furthermore, it can be observed from Table 4 that modifying the backbone has a beneficial impact on the detection performance of the YOLOv9c model.

**TABLE 6:** Model parameters and size floating point operation magnitude.

Model name	Layers	Parameters	GFLOPs
YOLOv5n	157	1,761,871	4.2
YOLOv5s	157	7,015,519	15.8
YOLOv5m	212	20,856,975	48.0
YOLOv5l	267	46,113,663	107.8
YOLOv7	314	36,487,166	103.3
YOLOv7t	208	6,010,302	13.1
YOLOv9t	658	2,617,340	10.7
YOLOv9s	658	9,598,796	38.7
YOLOv9m	588	32,554,612	130.7
YOLOv9c	604	50,700,588	236.6
YOLOv9c‐Faster	580	43,709,136	196.2

**FIGURE 6:**
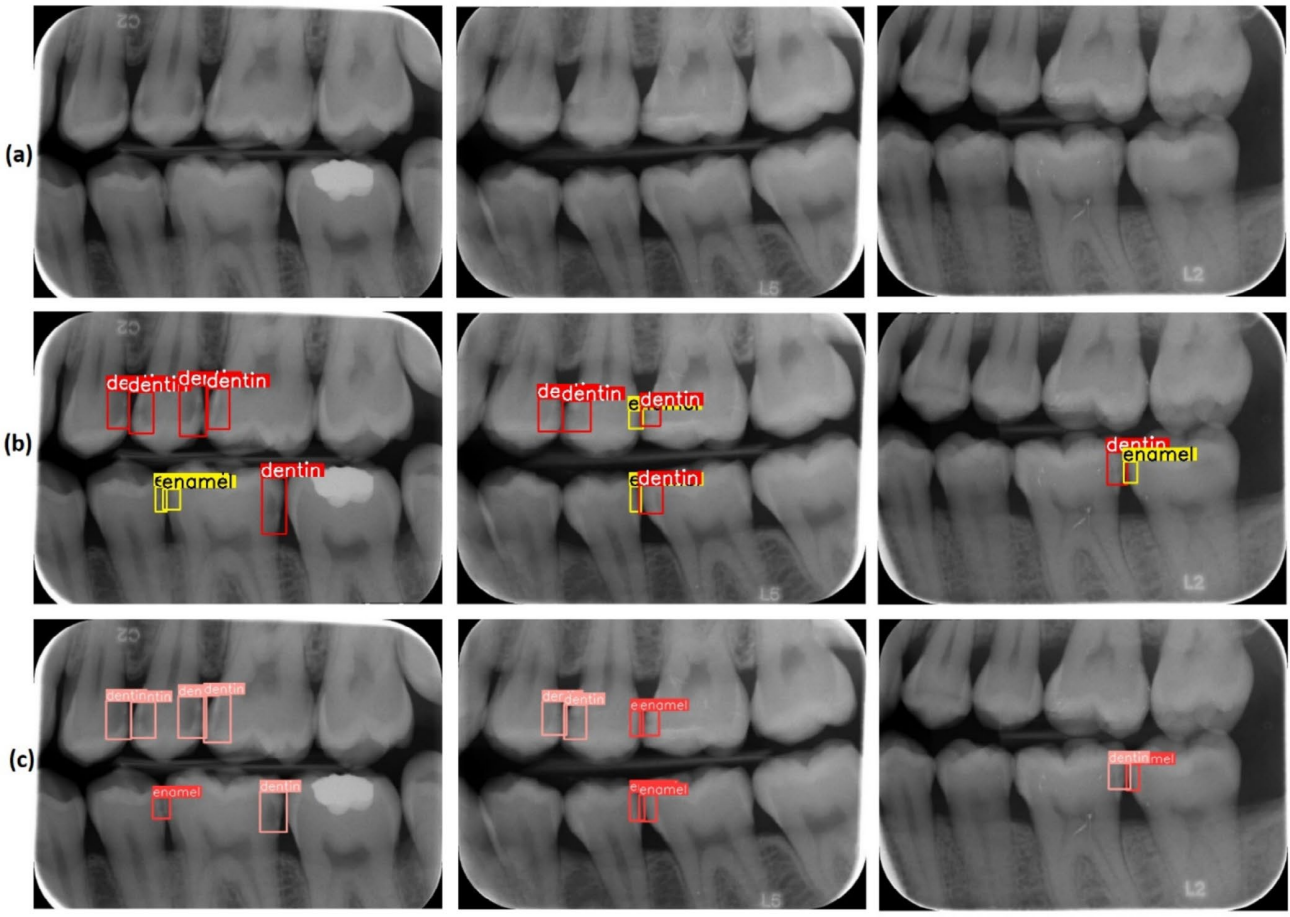
Some test samples from the dataset by model (a) original image, (b) ground truths, (c) model predictions.

Figure 6 presents some examples based on the predictions of the model. According to Figure 6, the model has generally achieved results consistent with the ground truths. However, in some cases, it has been observed that the model made inaccurate or incomplete predictions; particularly, the accuracy needs to be improved in small enamel regions. Overall, the model has demonstrated successful performance in dental caries detection, though further optimization is required to improve accuracy in smaller areas.

Despite the promising performance observed under the controlled conditions of this study, several challenges remain regarding its translation to clinical practice. While the model exhibited high accuracy within the confines of this investigation, its real‐world application is complicated by the inherent variability in radiographic quality and patient‐specific factors, which may impact diagnostic efficacy. The dataset employed herein underwent rigorous curation; however, dental radiographs are known to exhibit considerable heterogeneity in terms of image quality, exposure parameters, and the presence of artifacts. Future research should focus on developing models that can generalize effectively across diverse radiographic modalities and variable image quality, a vital step toward seamless integration into routine clinical workflows. Additionally, efforts should prioritize expanding datasets to encompass a wider variety of caries types and enhancing model interpretability to maximize their clinical applicability.

## 5 Conclusion

According to the results obtained from this study, improved deep learning‐based models showed promising scores in the diagnosis of enamel and dentin caries on bitewing radiographs. The use of such models in caries detection offers the potential to improve diagnostic accuracy, reduce workload, and ultimately enhance patient care. Future studies are required to focus on improving caries detection models by enhancing data quality and diversity through the use of larger, well‐annotated datasets and minimizing false positives and false negatives through advanced optimization techniques.

## Ethics Statement

All procedures performed in studies involving human participants were in accordance with ethical standards of the institutional and/or national research committee and with the 1964 Helsinki Declaration and its later amendments or comparable ethical standards. The study was approved by the Ethical Committee of Kırıkkale University (Date: 31.01.2024, Decision Number: 2024.01.27).

## Consent

The authors have nothing to report.

## Conflicts of Interest

The authors declare no conflicts of interest.

## Data Availability

The data that support the findings of this study are available on request from the corresponding author. The data are not publicly available due to privacy or ethical restrictions.
